# Feasibility of BCI Control in a Realistic Smart Home Environment

**DOI:** 10.3389/fnhum.2016.00416

**Published:** 2016-08-26

**Authors:** Nataliya Kosmyna, Franck Tarpin-Bernard, Nicolas Bonnefond, Bertrand Rivet

**Affiliations:** ^1^Team Hybrid, InriaRennes, France; ^2^Team EHCI, Université Grenoble AlpesSt Martin D'Heres, France; ^3^Inria MontbonnotMontbonnot St Martin, France; ^4^Gipsa Lab, Grenoble INPGrenoble, France

**Keywords:** brain computer interfaces, smart home, usability, conceptual imagery, EEG

## Abstract

Smart homes have been an active area of research, however despite considerable investment, they are not yet a reality for end-users. Moreover, there are still accessibility challenges for the elderly or the disabled, two of the main potential targets for home automation. In this exploratory study we design a control mechanism for smart homes based on Brain Computer Interfaces (BCI) and apply it in the “Domus”^1^ smart home platform in order to evaluate the potential interest of users about BCIs at home. We enable users to control lighting, a TV set, a coffee machine and the shutters of the smart home. We evaluate the performance (accuracy, interaction time), usability and feasibility (USE questionnaire) on 12 healthy subjects and 2 disabled subjects. We find that healthy subjects achieve 77% task accuracy. However, disabled subjects achieved a better accuracy (81% compared to 77%).

## Introduction

Imagine you could control everything with your mind. Brain Computer Interfaces (BCIs) make this possible by measuring your brain activity and allowing you to issue commands to a computer system by modulating your brain activity. BCIs can be used in many applications: medical applications to control wheel chairs or prosthetics (Wolpaw et al., 2002) or to enable disabled people to communicate and write text (Yin et al., 2013); general public applications to control toys (Kosmyna et al., 2014), video games (Bos et al., 2010) or computer applications in general. One of the more recent fields of applications of BCIs are smart homes and the control of their appliances. Smart homes allow the automation and adaptation of a household to its inhabitants. In the state of the art of BCIs applied to smart home control, only younger healthy subjects are considered and the smart home is often a prototype (single room or appliance). BCIs have never been applied and evaluated with potential end-users in realistic conditions. However, smart homes are of the interest to disabled people or to elderly people with mobility impairments who are able to operate appliances within the house autonomously (Grill-Spector, 2003; Edlinger et al., 2009). Studies on disabled users are just as rare as studies in realistic smart homes (with healthy subjects or otherwise). However, the expectations and needs of healthy subjects are biased, as they cannot fully conceive of the difficulties of disabled people and thus of their needs, so performing experiments both with healthy and disabled subjects is of interest for smart home research.

The purpose of the work is to evaluate the feasibility of BCI control in three steps:

1. Evaluate the feasibility of BCI control in a realistic smart-home environment in a state of the art setting on healthy subjects as an extrapolation of the potential results for disabled subjects. We propose an appliance control (toggle—light, TV, water kettle, shutters) scenario that allows us to evaluate task accuracy and performance. Then, we administer a usability (USE) questionnaire:
  a. Usefulness: how useful the interaction is with relation to the tasks?
  b. Ease of use: how easy to operate the control modality is (e.g., does the interface do what I want as I want it without significant effort)?
  a. Ease of learning: how easy were the concepts and operation instructions to grasp?
  b. Satisfaction: was the control provided satisfactory with relation to the expectations of users?
2. We validate the actions proposed in the scenario to ensure their adequateness with the potential needs of the subjects (Naturalness, Ease of Conceptualization and Adequateness to Expectations questionnaire).
3. Evaluate the feasibility of BCI control in the same experimental setting and protocol with subjects suffering from motor disability.

For experiment 1, our hypothesis (H1) is that BCI control will obtain task accuracy within the range of the state of the art and (H2) that the control modality will be liked and accepted by users (above 5 on the questionnaire scale).

For experiment 2, we hypothesize (H3) that BCI accuracy and performance will be at least as good as for experiment 1, or even slightly better as there is potentially less interference from muscle movement.

### Conceptual brain computer interfaces (BCIs)

Conceptual imagery is a recent development, where the aim is to capture Electroencephalography (EEG) in order to detect when the user thinks of a category of conceptual objects (hammer/tool, cat/animal) by Simanova et al. (2010). They considered textual, visual and auditory cues. They find that the best performing BCI system resulted from visual stimuli. The stimuli featured abstract or concrete images of objects from various categories that the BCI proceeded to recognize. Thus, conceptual imagery constitutes a powerful type of BCI that can lead to more natural interactions for users by using the semantics of the task. For example, it was shown by Carmel and Bentin (2002), Grill-Spector (2003), and Itier and Taylor (2004) that different classes of stimuli, such as cars, mushrooms, chairs, shoes, animals, etc., evoke spatially and temporally different responses, so one could imagine/visualize a key or a car itself to trigger the ignition of a car.

#### EEG, event related potentials, and functional magnetic resonance imaging to measure object categorization

Neuroscientific research has shown that when a user performs a goal-oriented action, the cortical activation related to object recognition and association are different depending on whether the presented object is the source or target of an action (Gallese et al., 1996; Helbig et al., 2006). Object recognition studies have shown that recognition rates improve when users are exposed to realistic representations of objects (Gallese et al., 1996) (scale, size, appearance). The best way to ensure a good representation of the objects (target or source) during training is to give users tangible feedback on the actual objects in the environment.

In their work, Shenoy and Tan (2008) explore whether the Event Related Potential (ERP) features that encode object category can be used to label images on a single-trial basis, without explicitly requiring users to consciously categorize the images. They perform an experiment where they show three different categories of images: faces, inanimate objects and animals, where the discrimination was achieved at more than 65 percent of accuracy. They did not control or eliminate any of the traditionally considered noise elements (e.g., 60 Hz power hum, etc.) found in the experimental environment, in order to simulate an environment that was likely both in a lab, as well as in a real-world setting. Before beginning, the EEG device was explained to the participants of their experiment and requested that they try to reduce unnecessary physical movements during the testing phases of the experiment. This, however, was not enforced. Another work by Kosmyna et al. (2015a) explores whether it is possible to build an asynchronous ERP-based BCI to distinguish between objects of similar and dissimilar conceptual categories and reaches classification accuracies around 65% for similar conceptual categories and around 70% for conceptually distinct categories.

Functional Magnetic Resonance Imaging (fMRI) studies of conceptual discrimination, reveal characteristic activations of certain regions of the brain, namely, Haxby et al. (2001) showed that the representation of an object is reflected by a distinct pattern of response across all ventral cortices and that this distributed activation produces the visual perception. They performed an experiment where the activation patterns for eight object categories were replicable such as faces, cats, houses, chairs, scissors, shoes, bottles, and nonsense pictures. Moreover their analysis indicated that it is possible to predict the category of the object even when regions that show maximal activation to that particular category are excluded. Spiridon and Kanwisher (2002) further show that such patterns are replicable even when the object format is changed and when the objects are shown from a different viewpoints. The evidence from brain imaging studies indicates that the discrimination between objects is possible with fMRI and to a limited extent with EEG (visually) with an acceptable degree of discrimination.

### Smart homes and BCIs

There are several works that use BCIs in smart home environments. A good part of those take place in virtual smart home environment (VR in Table 1) as opposed to a real or prototyped smart home (Prototyped Smart Home in Table 1). This can be problematic as it is difficult to recreate *in situ* conditions *in-vitro* (Kjeldskov and Skovl, 2007). There are several types of BCI control:

- *Navigation* (virtual reality only): The BCI issues continuous or discrete commands that make the avatar of the user move through the virtual home. Motor imagery (MI), where users imagine moving one or more limbs to generate continuous control is the most commonly used BCI for navigation.
- *Trigger/Toggle*: The BCI issues a punctual command that triggers a particular action or toggles the state of an object in the house (e.g., looking at a blinking led on the wall to make the light turn on). Many paradigms can be used for such actions:
- P300: The user looks at successively flashing items to choose from. When the desired item flashes, the brain produces a special signal (P300) that can be detected.
- Steady-State Evoked Potentials (SSVEP): The user looks at flickering targets at different frequencies. We can detect the target the user is looking at and trigger a corresponding action.
- Facial Expression BCI: The facial expressions of the user are detected though their EEG signals.

**Table 1:** **Summary of related work pertaining to BCI controlled smart homes**.

**Paper**	**Environment + paradigm**	**Classes**	**Subjects**	**Avg. BCI Perf**.	**User opinion**	**Time**
Carabalona et al., 2010	Prototyped smart home P300 (*trigger*)	Light, radio, bell	4 subjects with motor disability	Chr. Speller = 65% Icon Speller = 45.5%	Extr. positive (6 ot 7 out of 7) or Extr. negative (1 or 2 out of 7)	N/A
Simoens et al., 2014	Prototyped smart home|facial expression + BCI (*trigger*)	BCI activation of arbitrary objects selected by a camera	Not mentioned	No evaluation, theoretical proposition only	N/A	N/A
Edlinger et al., 2009	VR smart home | P300 (*select* / *trigger*)	30 actions in a grid (e.g., turning on the TV or the lights, selection of TV programs)	38 healthy subjects (offline) 3 healthy subjects in VR smart home	P300 = 82%	N/A	6 min of training. No session time
Edlinger et al., 2011	VR smart home | P300 (*selection*) + SSVEP (*trigger*)	30 actions in a grid (e.g., turning on the TV or the lights, selection of TV programs)	3 healthy subjects	P300 = 100% With SSVEP: 4.68s activation time Without SSVEP: 5.40s	N/A	30 min session. Same training time as above.
Su et al., 2011	VR smart home | P300 (*toggle*) + MI (*navig.*)	2 Left/Right + 3 song choices, quit, stop	4 healthy subjects	Across Task Avg P300 = 81.68% Across Task Avg MI = 84.54%	N/A	Avg. Task time MI = 58.70s Avg Task time P300 = 59.20s

Avg. BCI Perf., Average BCI performance; Chr, Character; Extr., Extremely.

Table 1 summarizes state of the art work that uses BCIs to control smart homes, following the aforementioned aspects. We present the reference of the work, the nature of the environment (virtual or real smart home), the number and type of actions/classes controlled by the BCI and the BCI paradigm. We indicate the average BCI accuracy, the user opinion evaluation and experiment/training time where this information is available.

In this work we use only trigger-type controls. Most BCIs applied to smart homes use paradigms (P300, SSVEP) that require a display with flashing targets that the user must look at. Whereas, here, we use conceptual imagery, that requires no external stimuli. Conceptual imagery has never been applied to any practical control application, let alone smart homes. With conceptual imagery the semantics of the interaction is compatible with the semantics of the task. The principle is that users imagine the concept of a lamp (e.g., by visualizing a lamp in their mind) and the BCI recognizes the concept and trigger a command that turns on the light. The concepts exactly correspond to the appliances to actuate (See **Figure 5**).

Although the experiments of the state of the art for smart home control are numerous, the added value of our experiment is the use of a paradigm that better matches the semantics of the task users had to perform. In other BCI experiments, the control was mainly performed with paradigms such as P300 or SSVEP that require the production of external stimuli that users have to look at in order to select the desired command. This requirement is cumbersome and poses serious constraints outside the mainly virtual smart homes where such paradigms have been tested. This approach is akin to a remote control that is completely separate from the task. The cases where Motor Imagery could be used in a semantically relevant way are restricted to the gradual actuation of some devises, but would hardly allow a semantically relevant selection. Conceptual imagery on the other hand matches well with the semantics of a selection task. One visualizes the appliance and it is selected/activated.

## Materials and methods

### Design of the smart home

#### “domus” platform

**Figure 1:**
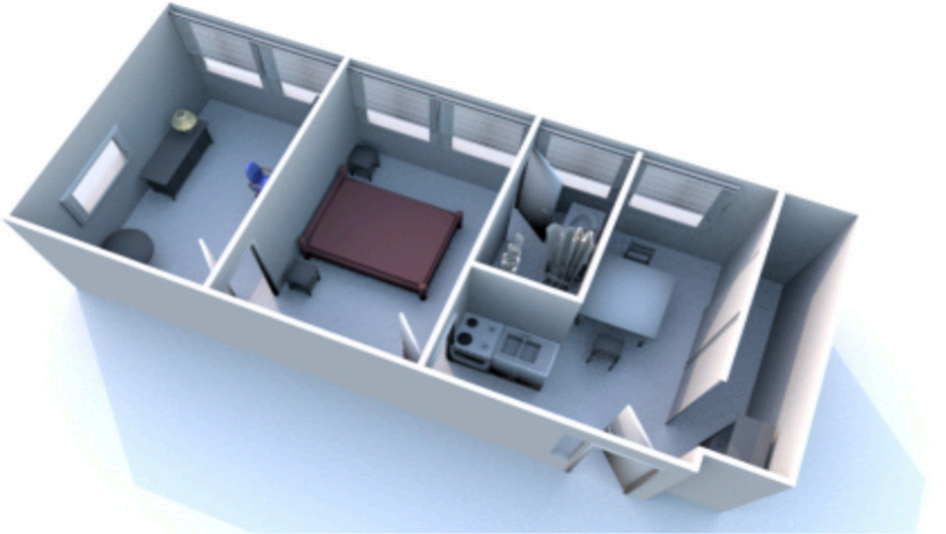
**The “Domus” Smart Home**.

The “Domus” smart home^2^ is part of the experimentation platform of the Laboratory of Informatics of Grenoble (Figure 1). “Domus” is a fully functional 40 meters square flat with 4 rooms, including a kitchen, a bedroom, a bathroom and a living room. The flat is equipped with 6 cameras and 7 microphones to record audio, video and to monitor experiments from a control room connected to “Domus.”

##### Appliances

The flat is equipped with a set of sensors and actuators using the KNX^3^ (Konnex) home automation protocol.

The sensors monitor data for hot and cold water consumption, temperature, CO2 levels, humidity, motion detection, electrical consumption and ambient lighting levels. Each room is also equipped with dimmable lights, roller shutters (plus curtains in the bedroom) and connected power plugs that can be remotely actuated.

For example, in this experiment, in order to turn the kettle on or off, we directly control the power plug rather than interacting with the kettle. 26 led strips are integrated in the ceiling of the kitchen, the bedroom and the living room.

The color and brightness of the strips can be set separately, for an entire room or individually using the DMX protocol^4^.

The bedroom is equipped with a UPnP enabled TV located in front of the bed that can be used to play or stream video files and pictures.

##### Architecture

The software architecture of “Domus” is based on the open source home automation software openHAB^5^. It allows monitoring and controlling all the appliances in the flat with a single system that can integrate the various protocols used. It is based on an OSGI framework^6^ that contains a set of bundles that can be started, stopped or updated while the system is running without stopping the other components. The system contains a repository of items of different types (switch, number, color, string) that stores the description of the item and its current state (e.g., ON or OFF for a switch). There are also virtual items that exist only in the system or that serve as an abstract representation of an existing appliance function. The item is bound to a specific OSGI bundle binding that implements the protocol of the appliance, allowing the system to synchronize the virtual item state with the physical appliance state. All appliance functions in “Domus,” such as power plug control, setting the color of the led strips or the audio/video multimedia control are represented as items in the system. The event bus is also an important feature of openHAB. All the events generated by the OSGI bundles, like a change in an item state or the update of a bundle are reported in the event bus. Sending a command to change the state of an item will generate a new event on the bus. If the item is bound to a physical object, the binding bundle will send the command to the appliance using the specific protocol, changing its physical state (e.g., switching the light on). The commands can be sent to an item through HTTP requests made on the provided REST (Gallese et al., 1996) interface. A web server is also deployed in openHAB, allowing the user to create specific UIs with a simple description file containing the items to control or monitor (**Figure 4**). The user can also create a set of rules and scripts that react to bus events and generate new commands. Persistence services are also implemented to store the evolution of states of items in log files, databases, etc.

##### Control capabilities

A virtual item was created in the repository to represent the classification produced by the BCI and speech recognition systems. A set of rules was also set to be triggered when this virtual item state changed. Depending on this new state and the current room where the user is located, the rule sent a command to the specifics items to control the physical appliances associated to classification outcomes (TV, kettle power plug, etc.). To control the virtual item, the BCI/speech programs communicated with openHAB using the provided REST API. After a classification was performed, a single http POST request containing the classification outcome a string was sent to the REST interface, changing the state of the virtual item, triggering the rule and therefore allowing the user to control the desired appliance with the EEG headset.

### Our brain-computer system implementation

**Figure 2:**
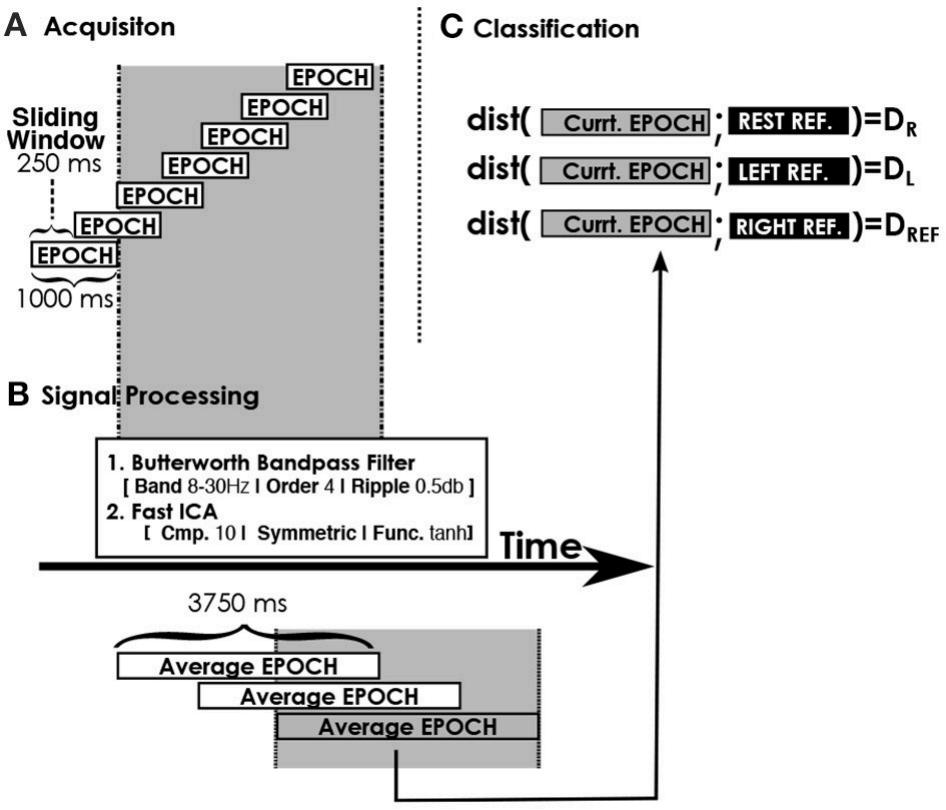
**An illustration of the functioning of the BCI system and the classifier**.

We have used an asynchronous BCI system based on a Minimum Distance Classifiers (MDCs) that requires the recording of at least one reference per BCI class in order to function. It has shown competitive performance compared to state of the art systems while being simple and fast (Barachant et al., 2012). Moreover its simplicity and low computational requirements allow for classifications to occur at short intervals, which is particularly useful for real-time use (e.g., in a smart home). Figure 2 illustrates the functioning of our system. The raw signals are chunked into 1s epochs that overlap over 250 ms. They are then filtered to retain frequency bands relevant for conceptual imagery [we apply a Butterworth pass band filter between 4 and 30 Hz following the set-up of Simanova et al. for their pilot study on conceptual imagery (Simanova et al., 2010)] and decomposed in components that separate noise from actual useful signals. We use Fast ICA with 10 iterations (10 components extracted out of 16 sensors) in a purely unsupervised manner. The aim is to separate artifacts and noise from the rest of the activity in order to improve the accuracy of the distance measure that would normally be biased by such noise. Then, the epochs are averaged 5 by 5. The classifier is applied on each of the average signals by computing a distance between each reference and the current average epoch and by picking the class with the minimum distance. The rationale behind the system and its design choices are presented by Kosmyna et al. (2015b). When a classification is performed, the command is immediately sent to the smart home and is executed.

#### Hardware

**Figure 3:**
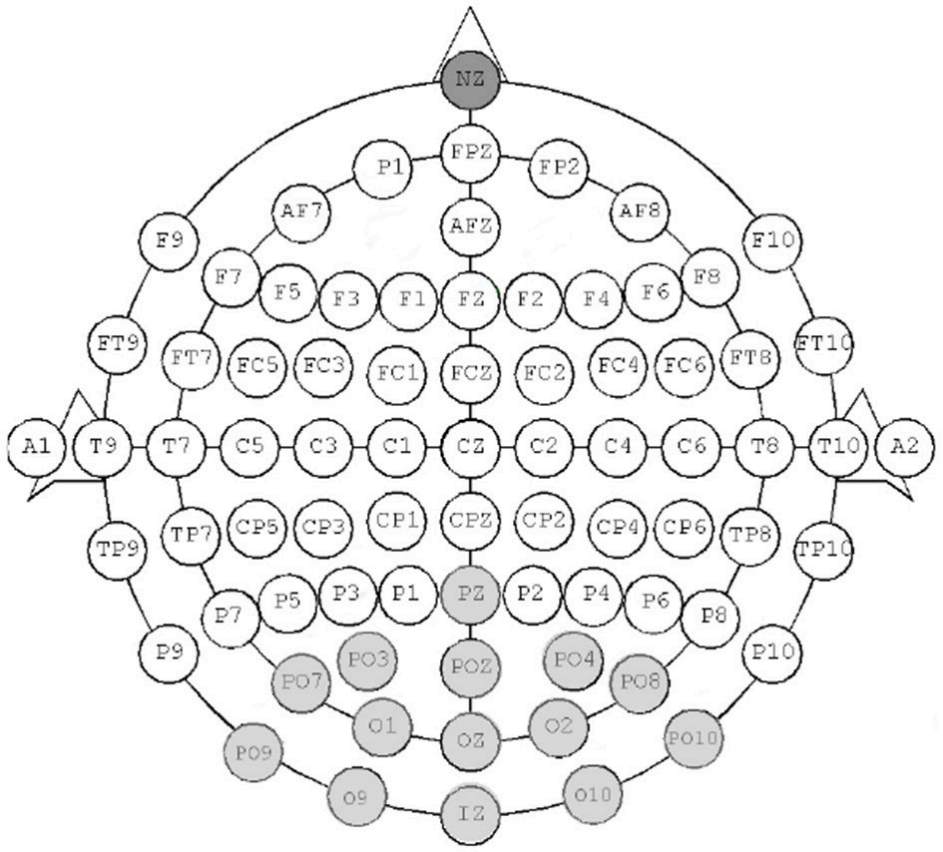
**The electrode placements for conceptual imagery used for the g.tec USBAmp**. The dark gray electrode is the reference, the light gray ones are the CI electrodes.

We used the g.tec USBAmp, a high-end bio signal (EEG, Electrocardiography or EKG, others) amplifier. We use it coupled with 16 electrodes on a standard 10–20 EEG cap. We use a sampling frequency of 512. Figure 3 shows the electrode placement for conceptual imagery. We placed the electrodes only over the visual cortex, as it was shown by Simanova et al. (2010) that the discrimination of conceptual categories was the best over the visual cortex (corresponding the visual imagination of the concepts) and as we followed a similar procedure in order to maximize the classification accuracy.

#### BCI feature validation

In order to verify the quality of the BCI system prior to the experiments and validate the system, we performed a simple validation. We classified a set of unlabeled signals and compared them to a set of reference signals for each class using 10-fold cross-validation. We used the three distance measures that can be used with our system in order to see what is the best distance, if there is one. The analysis was done over the signals of two subjects captured over the course of 10 sessions. We can now look at the cross-validation results from the off-line analysis to validate the BCI system depending on the distance measure used in Table 2. In bold is the best result for each measure and each subject. We did not find that any of the distances had an absolute advantage over the others: it varies from subject to subject. Thus, we decided to add a control that allows dynamically switching the distance measure used during the online phase so that it could be easily adapted to each user.

**Table 2:** **10-fold cross-validated accuracy of our BCI system on two subjects over 10 sessions for Conceptual Imagery (CI)**.

	**Dist. measure**	**Accuracy Subject A**	**Accuracy Subject B**
CI	Maha.	**74.24% (2.54%)**	73.35% (1.42%)
	Riem.	73.45% (1.51%)	**74.26% (2.97%)**
	Corr.	72.92% (2.10%)	**73.34% (1.23%)**

### Usage scenarios

The actions to be performed in the home as well as the scenarios themselves are based on the responses of the participants in a 1-year long-term study (Brush et al., 2011). Almost all of the users of the study would like to have had a possibility to:

- Adjust windows and shades automatically to keep house comfortable;
- Turn devices on/off based on presence.

We propose to use our BCI system (Experiment 1 and 2) so that the user can voluntarily:

- Turn the kettle on and off;
- Lower or lift the shutters;
- Turn the TV on or off;
- Turn the light on or off.

**Figure 4:**
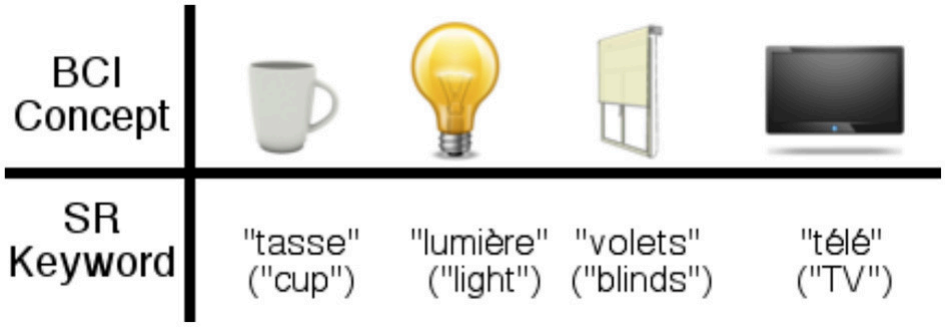
**Visual stimulations used for each concept for the conceptual BCI control**. The cup toggles the kettle on or off, the lamp toggles the lights on or off, the blinds toggle the blinds to be raised or lowered and the TV triggers the TV on or off.

For the BCI control (Experiments 1 and 2), the users had to imagine a concept associated to each action. We used four images during the BCI training (Figure 4, light, cup, shutters, TV).

We use two of the three rooms in “Domus,” the kitchen and the bedroom.

We describe the scenario that the users had to perform below and show some corresponding photographs for a BCI interaction.

1. Enter the “Domus” smart home.
2. Turn on the light.
3. Move two steps toward the table with the kettle.
4. (Wait 10–15 s without moving) Turn on the kettle.
5. Wait for around 1 min.
6. Stop the kettle.
7. Move to the bedroom and lay down on the bed.
8. (Wait 10–15 s without moving) Lower the blinds.
9. Turn on the TV set and watch it for around 5 min (BCI deactivated during this period).
10. Turn the TV set off.
11. Raise the blinds.
12. Go back to the kitchen.
13. (Wait 10–15 s without moving) Turn off the light.
14. Exit the “Domus” smart home.

### Experimental protocol

**Figure 5:**
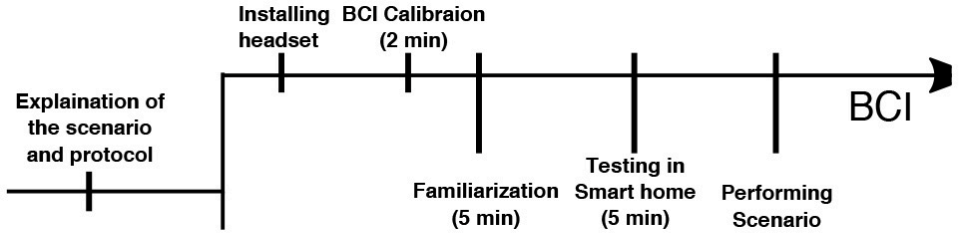
**Timeline for the experiments**. “Familiarization” phase let the users test the BCI without any actuation of the smart home appliances. The “Testing in smart home” phase is the same as familiarization, except the users had the actuation active.

1. We first briefed the users to the layout of the smart home and explained the various interactions to be performed as well as the details of the experiment (Figure 5, step 1).
2. We installed the BCI headset on the users and gave safety instructions (Figure 5, step 2).
3. The users underwent the BCI training. Prior to the training instructions were given about the operation of the BCI. Then the users were trained using a synchronous training protocol (Figure 5, step 3).
4. We let the users roam free in “Domus” for 5–10 min to test the actuation of the appliances physically with the usual switched. For example, it happened that the users were startled the first few times when the blinds were lowering; as the motor emits a noise they are unaccustomed to (Figure 5, step 4, Familiarization).
5. Then, we activated the BCI and let users test the actuation of the appliances using the BCI freely (Figure 5, step 5, Testing in smart home).
6. Then, each user performed the scenario described in the previous section (Figure 5, last phase).

**Figure 6:**
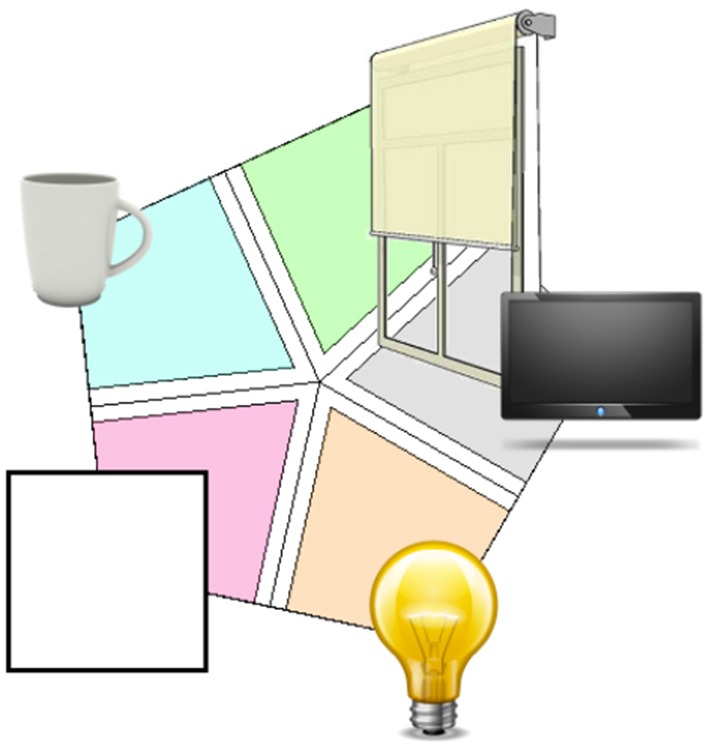
**Calibration of the toggle blinds class**.

There are 5 BCI classes (the 4 commands + Resting state). Each user had to go through a 2 min initial training for the BCI + 5 min of familiarization with the BCI + 5 min of testing in the Domus before the session started). Prior to training, we briefed the user for 5 min on imagination strategies for the BCI and recommended that they imagine the object and visualize it a practical setting. During the BCI training the classes are presented in a variant of radial class visualization and each class is successively magnified to indicate that users must imagine that particular object (Figure 6). Before the testing phase we instructed subjects to maintain the same imagination strategy as during training.

We installed the g.tec cap on the user prior to the experiment. We instructed users to stop moving for at least 5 s when they started imagining the concept to trigger in order to minimize interference from movement.

For the evaluation, we use task accuracy that we calculate as such: for each underlined step in the scenario, 5 s after the user stopped moving; we started counting the number of classification outcomes different from the expected result until the expected class was obtained. We totalled the number of true positives, true negatives, false positives, and false negatives and computed the accuracy over one full scenario for each subject ((TP+TN)/(TP+FP+FN+TN)). For each of the experiments we present the overall median values. We also measured Interaction time, on average for each task and the scenario completion. The 10–15 s pauses in the step were to ensure that any residual activity from changing rooms has subsided and lets users return to a resting state before using the BCI.

After the experiment, we asked the users to fill the USE questionnaire proposed by Carabalona et al. (2010) that was used for the evaluation of the smart homes as well. The questionnaire contains a series of questions about their experience graded on Likert scale from 1 to 7. The questions were categorized among four criteria: Usefulness, Ease of use, Ease of learning, Satisfaction.

### Experimental population

The usual demographic for BCI control are disabled subjects who can use no other modalities for control. However, there are valid reasons to first evaluate the control on healthy subjects. Several studies outline that healthy users are acceptable for preliminary investigation granted conditions are met (Sears and Hanson, 2011). “Healthy users should be artificially put in a state of situational disability” (Allison, 2010; de Negueruela et al., 2011), where they are not advantaged compared to disabled subjects. Similarly, in Bigham and Cavender (2009) healthy and blind users are used indiscriminately, however healthy subjects are put in situational disability by being blindfolded. This is also true of BCI systems for Smart Homes in VR environments evaluated on healthy subjects (Huang, 2006; Edlinger et al., 2011) (See Table 1).

Thus, we first select 12 subjects aged between 23 and 45 in good physical and mental health for Experiment 1. They all had BCI experience (to counter for novelty bias). None had any smart-home experience.

Then we considered two subjects with motor disability in a wheel chair and had them perform the same scenario with the BCI for Experiment 2. Both had prior BCI experience. Subject 1 was 27 year old and used a manual wheel chair. This subject had occasional leg tremors and some brief moments of limb restlessness. The subject was otherwise in good health. Subject 2 was 25 years old and had an electric wheelchair. He had no other discernable health issues.

### Validation of the commands

For the statistical validation of the results, we first checked the normality of the data, for the first experiment, the equality of variances is checked with the Levene test between all pairs of variables and the null hypothesis is confirmed with *p* < 0.01. The Shapiro-Wilk test for each of the variables led to *p*-values higher than 0.01, thus validating that the null-hypothesis of the non-normality of the data can be rejected. The results are thus presented as the mean for each measure and each controlled object and the error bars are the 95% Confidence Interval for the normal distribution. We used the same procedure as for the validations of the commands to check for the normality of the distributions of each measure.

**Figure 7:**
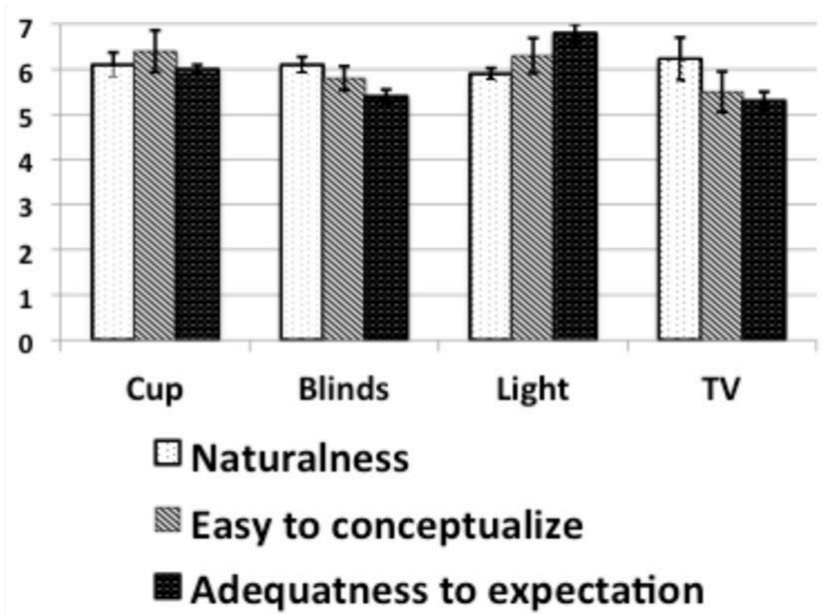
**Averaged answers given by users about the naturalness of the action, the easiness of conceptualization and the adequateness to expectation on a scale from 1 to 7**. The error bars are the 95% confidence interval.

Before performing the experiments, we explained the principle to users and the different actions the BCI would allow them to perform. After each session, we gave them a questionnaire to asses whether the particular actions were natural, easy to conceptualize and adequate with relation to their expectation. We devised the questionnaire so as to have several questions for each of the three aspects and for each class. The questions were based on a 7-point Likert scale. Questions were in the form of affirmative statements such as “I found the action of turning the TV on/off natural.” Figure 7 shows the averaged answers given by users about the naturalness of the action, the easiness of conceptualization and the adequateness to expectation. We can see that for all the actions all three indicators scored close or above 6, which indicates that the action chosen are appropriate to what users would expect.

## Results

While, for healthy subjects, the distributions were normal, it was not the case for the disabled population due to low samples while testing for differences between healthy and disabled users. We used a non-parametric test to check for the pairwise significance of the difference between the medians (Mann-Whitney U). The results are presented as medians and the error bars are 95% bootstrapping confidence intervals.

Let us now examine the results for the first experiment that stem from the questionnaires along the four axes of Usefulness, Ease of Use, Ease of Learning and Satisfaction.

**Figure 8:**
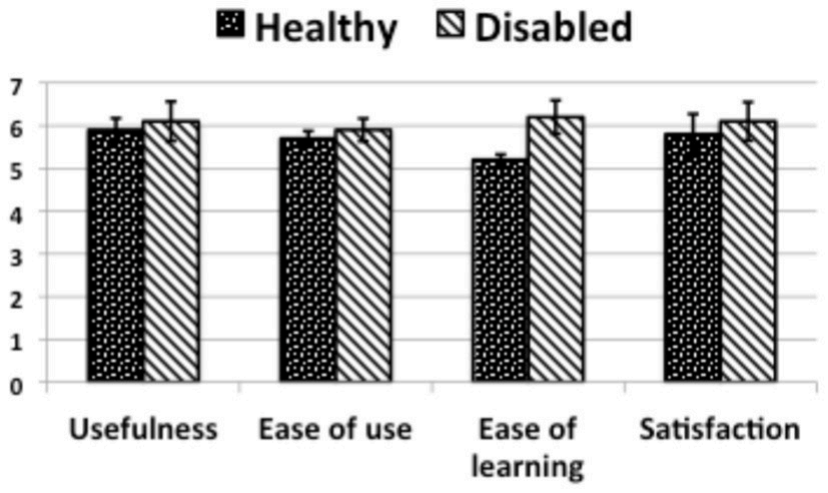
**A bar chart summarizing the median answers of the user for each question category across Experiment 1 and 2**. The error bars are the 95% bootstrapping confidence intervals.

**Figure 9:**
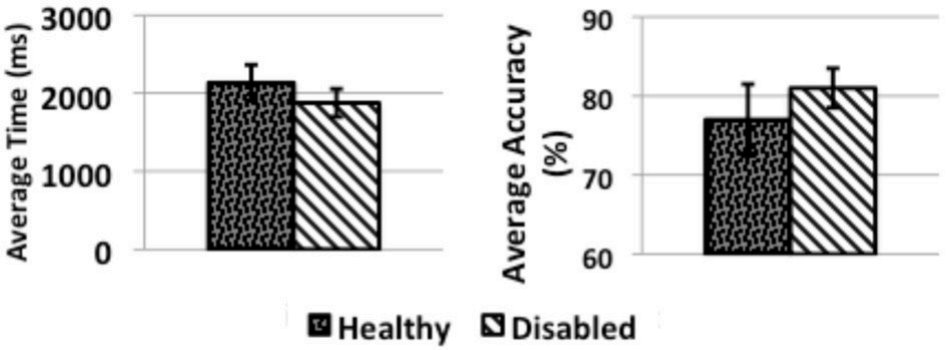
**Bar charts summarizing the median task accuracy and time to activation across Experiments 1 and 2**. The error bars are the 95% bootstrapping confidence intervals.

Figure 8 (“Healthy”) presents the quantitative summary of the answers to the questionnaire in the four categories for the experiment with healthy subjects. The results are the mean of the scores answered to questions in each category on a scale from one to seven. Usefulness, ease of use, ease of learning and satisfaction scored high, with slightly less than 6/7 on average. Moreover, the BCI obtained a task accuracy of 77% and a mean correct activation time of 2 s, which places it in the range of state of the art performance for BCI systems intended for smart home control (Figure 9, “Healthy”).

We also informally asked users to make comments regarding both settings. Often mentioned negative points for the BCI system were: “The training requires mental effort”; “The classification was not always correct.” On the other hand there were positive remarks that it was “amusing” or that “Using my brain to control things in a house is really cool, it would be great if I could do the same at home.”

For the questionnaire results (Figure 8 “Disabled”) of the second experiment, in terms of usefulness, ease of use and satisfaction there were no significant differences with healthy subjects (slightly above 6/7). Ease of learning was rated significantly higher than for the healthy subjects (*U* = 9 < 11, the critical value for *p* = 0.05). The task accuracy (Figure 9 “Disabled”) was better with 81% instead of 77% for healthy users however the statistical test did not show the difference as significant (*U* = 34 > 11, the critical value for *p* = 0.05).

The subjects had positive reactions, “This BCI control was interesting and very different from what I'm used to.” They were satisfied with the amount of control they could achieve. Subject 2 suggested that, for example, being able to control the room temperature with the BCI would be something invaluable as at his home he has absolutely no way of doing this alone (“for me personally, I would replace the kettle by a switch for centralized heating, at home I can never operate it myself”).

## Discussion

In this study we designed a control mechanism for smart homes based on Brain Computer Interfaces (BCI) and apply it in the “Domus”^1^ smart home platform in order to evaluate the potential interest of users about BCIs at home. We enabled users to control lighting, a TV set, a coffee machine and the shutters of the smart home. We evaluated the performance (accuracy, interaction time), usability and feasibility (USE questionnaire) on 12 healthy subjects (experiment 1) and 2 disabled subjects (experiment 2).

The purpose of the experiments was to evaluate the feasibility of BCI control:

1. For experiment 1: to evaluate the feasibility of BCI control in a realistic smart-home environment in a state of the art setting on healthy subjects as an extrapolation of the potential results for disabled subjects.
2. For experiment 2: to evaluate the feasibility of BCI control in the same experimental setting and protocol with subjects suffering from motor disability.

For experiment 1, our hypothesis (H1) was that BCI control will obtain task accuracy within the range of the state of the art and (H2) that the control modality will be liked and accepted by users (above 5 on the questionnaire scale).

For experiment 2, we hypothesized (H3) that BCI accuracy and performance will be at least as good as for experiment 1, or even slightly better as there is potentially less interference from muscle movement.

We found that healthy subjects have achieved 77% task accuracy. However, disabled subjects achieved a better accuracy (81% compared to 77%).

Our hypothesis for this first experiment is thus verified (H1 and H2). If we compare these results with those in the state of the art (Table 1), we see that our results are in a comparable range (at most 4% difference) as the best state of the art results, despite the fact that in the state of the art the BCI training is much longer than for our system. Moreover, to the best knowledge of the authors, the inherent limitation of most smart home experiments with BCIs is that they are performed either in a virtual environment or at the prototype scale (Table 1). Few experiments take place in fully equipped smart homes *in-situ*. The added value of our research lies in the fact that the setting is an actual smart home environment.

For the second experiment, the results are not necessarily surprising. Disabled subjects appeared to be more motivated (subjective judgment) by the use of the BCI and were more focused both during training and during the performance of the task. The second subject even commented on the imagination strategy he was trying to use. This experiment gives some supportive evidence of H3, although the differences of medians are not statistically significant due the limited number of users in order to confirm H3.

The questionnaire results go on to show that unlike for healthy subjects, where there is no strong incentive to be able to control one appliance over another, disabled people do have to gain from a BCI control in such areas.

### Limitations

The training phase is short and it is crucial that it is done correctly, in order to ensure a good initial classification. This means that in some cases the system will need to be retrained several times in order to reach a stable performance. Moreover the classifier is sensible to noise, and movement can disrupt the performance of the BCI. Thus, it is required for users to wait at least 5 s before starting to attempt the interaction in order to ensure a good performance.

The number of controlled appliances is relatively low (although the classification accuracy is in line with the state of the art active BCIs for 4–5 classes), which is a limiting applicability factor. We could imagine an adaptive system that selects a subset of appliances depending on the location and orientation of the user to alleviate this issue or produce a BCI that can distinguish between more classes.

Although “Domus” is a full fledged home, fully equipped to enable people to live in it, our subjects were introduced to the environment at the beginning of the study and did not have to live there and experience the BCI over prolonged periods of time. Moreover the subjects knew they were going to use BCIs (perceived as cool) to control a smart home, something still out of science fiction for some people, even if they have already tried it once or twice. Given that our users are naïve and have never actually been in a smart home environment, this could lead them to be overly positive and thus to bias the answers to the questionnaires. The setting being an experiment (although in a realistic smart-home environment) and not real use in people's own homes, the user could also be influenced by what he or she thinks the expected conclusions are and to experience confirmation bias. As for disabled subjects, there is a need to perform experiments with more subjects (despite their scarcity) to validate the experiment.

### Future work

To truly validate the long-term acceptability of BCIs for smart home control we need a longer-term study with a cross-blind protocol. Using, as Barachant et al. (2012) suggest, a signal database to initialize the minimum distance classifier would lead to a better performance and make the use for end-users much more pleasant, this would also effectively remove the need to train the BCI. Although, some instructions to the user and a short calibration would be required, overall the time spent and tediousness on the part of the user would be significantly lower (comparable to operating an eye-tracker). Finally, here we preform experiments where the BCI is used for direct control, which is already possible with better and more robust modalities such as speech or gesture recognition. However, there are areas where BCIs can be used in a way that other modalities do no allow, for example in noisy environments where speech recognition may not be possible, or in low lighting conditions where gestures would not be practical.

## Conclusion

We performed a study over the control of a smart home with a BCI. We had users perform four toggle actions with the BCI. Healthy people exhibit performance similar to the state of the art in terms of BCI control and are satisfied by the control. However, disabled people achieved a better performance (81% compared to 77%). We conclude that while BCI is a suitable modality for use by healthy end-users except the comfort issues (wearing the headset), disabled end-users have stronger incentives and motivations to learn to use the BCI correctly. We conclude that using BCIs for smart home control is feasible but requires further study.

## Author contributions

The work was proposed, designed, and evaluated by NK. FT and BR were supervisors of the NK. The NB is an engineer in the Domus platform where the experiments took place, he helped the NK with the experiments (set-up of the platform, participants).

### Conflict of interest statement

The authors declare that the research was conducted in the absence of any commercial or financial relationships that could be construed as a potential conflict of interest.

## Footnotes

^2^http://domus.liglab.fr

^3^https://knx.org/knx-en/index.php

^4^http://www.dmx-512.com/dmx-protocol/

^5^http://www.openhab.org

^6^https://www.osgi.org
